# Validating species distribution models to illuminate coastal fireflies in the South Pacific (Coleoptera: Lampyridae)

**DOI:** 10.1038/s41598-021-96534-x

**Published:** 2021-08-30

**Authors:** Laura N. Sutherland, Gareth S. Powell, Seth M. Bybee

**Affiliations:** 1 Department of Entomology, Purdue University, 901 West State Street, West Lafayette, IN USA; 2 Department of Biology, Brigham Young University, 4102 LSB, Provo, UT USA; 3 Monte L. Bean Museum, Brigham Young University, Provo, UT USA

**Keywords:** Ecological modelling, Tropical ecology

## Abstract

The coastal areas of Vanuatu are under a multitude of threats stemming from commercialization, human development, and climate change. *Atyphella* Olliff is a genus of firefly that includes species endemic to these coastal areas and will need protection. The research that has already been conducted was affected by accessibility due to the remote nature of the islands which left numerous knowledge gaps caused by a lack of distributional data (e.g., Wallacean shortfall). Species distribution models (SDM) are a powerful tool that allow for the modeling of the broader distribution of a taxon, even with limited distributional data available. SDMs assist in filling the knowledge gap by predicting potential areas that could contain the species of interest, making targeted collecting and conservation efforts more feasible when time, resources, and accessibility are major limiting factors. Here a MaxEnt prediction was used to direct field collecting and we now provide an updated predictive distribution for this endemic firefly genus. The original model was validated with additional fieldwork, ultimately expanding the known range with additional locations first identified using MaxEnt. A bias analysis was also conducted, providing insight into the effect that developments such as roads and settlements have on collecting and therefore the SDM, ultimately allowing for a more critical assessment of the overall model. After demonstrating the accuracy of the original model, this new updated SDM can be used to identify specific areas that will need to be the target of future conservation efforts by local government officials.

## Introduction

The results of rapid industrialization (e.g., climate change, deforestation and light pollution) have a significant impact worldwide. An ability to quickly document and assess biodiversity in neglected and/or unknown areas of the world is paramount to biodiversity sciences and conservation. This need is compounded for biodiversity in isolated or unique areas of the world, where the habitat of endemics can be threatened^1,2^. A major challenge to biodiversity discovery, study, and potential conservation efforts is often the amount of area to cover, obtaining proper permits, limited funding, and time. There is an urgent need for tools that can focus biodiversity studies with little prior knowledge and/or input data.

**Figure 1:**
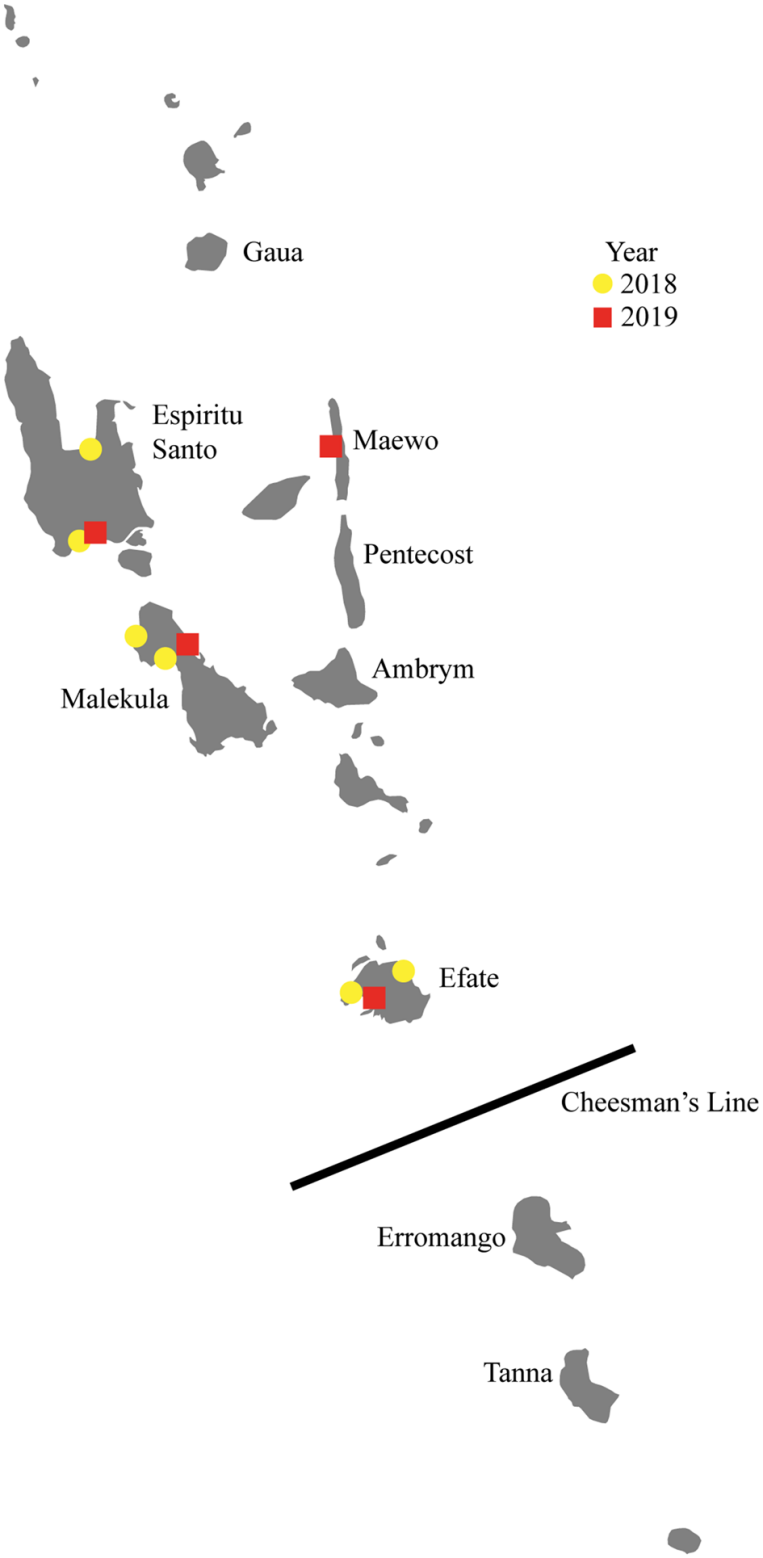
Distribution map showing occurrence data for *Atyphella* across Vanuatu based on field expeditions in 2018 and new locations from 2019. The islands explored over both years are labeled. Black line denotes the biogeographic break known as Cheesman’s Line (Vemaps.com, Adobe, San Jose, CA, USA).

Islands represent some of the most unique and unknown areas of the world while also being among the most, if not the most, fragile and threatened environments due to climate change, sea level rise, plastic pollution, and general human commercialization^3^. Islands also provide unique opportunities to investigate many biogeographical questions including fragmentation and biodiversity dynamics^4–6^. The South Pacific is an excellent region for biogeography, biodiversity, and conservation studies due to the number of islands and amount of variation within and between the archipelagos^7–9^. Specifically, Vanuatu is of interest because it is much younger (~ 2 million years old) than the surrounding island chains^5,9^. Vanuatu is comprised of 80 + islands, mainly of volcanic origin, with an approximate area of 12,190 km^2^ spanning 1300 km^9,10^. Vanuatu has a relatively lower documented species diversity due to the young age, size, and isolation, but is home to several unique and rare endemics^11^. These endemics are subject to potential dispersal barriers within the archipelago. The main barrier is the hypothesized biogeographic break, known as Cheesman’s line, between the islands of Efate and Erromango, separating the flora and fauna of the northern and southern islands (Fig. 1)^7,9,12^.

Vanuatu is an area of special interest to study coastline limited species due to the size, variation, and number of islands with varying coastline habitat. Across the four most diverse insect orders (Coleoptera, Hymenoptera, Lepidoptera, Diptera), accounting for almost 775,000 described species globally, species that are dependent on coast habitats are rare^13^. Specifically, within Coleoptera there are approximately 52 species from 13 families (< 0.01%) that are considered marine^14–16^. Doyen^15^ defined marine ranging from spending some time submerged by high tides to fully aquatic in the ocean. While many coastal species fall under this definition, we are defining coastal by restricting the definition of “marine” to those species that inhabit intertidal zone areas that are fully submerged during high tide.

Fireflies (Coleoptera: Lampyridae) are famous for their bioluminescence and there are ~ 2,250 described species^17^. Interestingly, there are several coastal species of firefly. *Micronaspis* Green has been collected from the Bahamas, Brazil, and Florida and there is a potential for a further intertidal species in Jamacia that remains unnamed^14,18,19^. *Atyphella* Olliff included a single coastal species*, A. aphrogeneia* (Ballantyne) that was found in Papua New Guinea and specimens were later collected in Vanuatu expanding their range and information known about their habitat requirements^14,20^.

**Figure 2:**
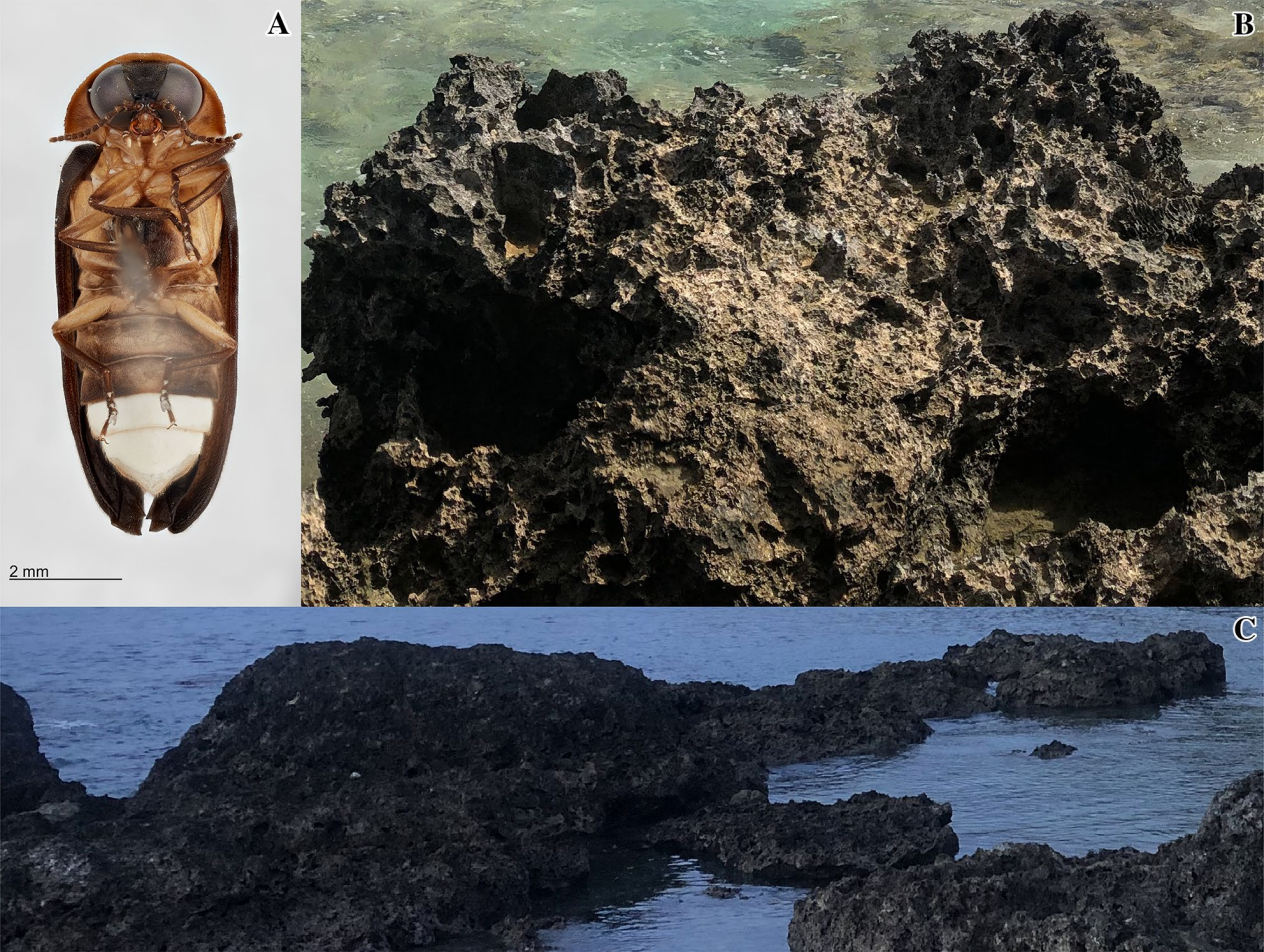
(**A**) Holotype of *A. maritimus*. (**B**,**C**) Photos of habitat, volcanic rock with extremely sharp and jagged edges lined the coast where *Atyphella* was collected. At high tide, the rocks were either mostly submerged or completely covered (Photo credit: (**A**,**B**) Colin Jensen and Natalie Saxton, (**C**) Saxton et al.^24^.

In the S.E. Asia and Australopacific region there are ~ 222 species which are split across 28 genera in Luciolinae^21^, with only three species now known to be coastal and they are all members of *Atyphella.* Until 2018, *A. aphrogeneia* was the only coastal species within this genus. Two additional species endemic to Vanuatu were discovered: *A*. *maritimus* Saxton and Powell and *A*. *marigenous* Saxton and Bybee^22^. *Atyphella maritimus* has only been collected from islands in the Malampa and Loyalty Basin bioregion while *A*. *marigenous* has only been collected from Efate which sits in the East Shefa bioregion, both species are exclusively found north of Cheesman’s line^22–24^. Both *A*. *maritimus* and *A*. *marigenous* can be observed at twilight with adult males flying over the intertidal zone and the larvae and females being found in the pockets and pocks of the sharp volcanic rock (Fig. 2)^14,24^. Larvae are often found below the water surface.

Species distribution models (SDM) of rare and endemic groups (i.e., *Atyphella*) are useful when there are logistic and/or financial constraints to survey specific areas for a species overall biodiversity (e.g., across Vanuatu). These models can function as powerful tools to direct field efforts to maximize the chance of locating, defining a range, and documenting biologically important data^25^. MaxEnt is one such program that uses environmental and climate data to determine criteria for a species niche and extrapolates it to produce a geographic range with an associated probability for each area^26–28^. MaxEnt is typically used for species level predictions, but it can be appropriate to use at a higher taxonomic level (e.g., genus) if all the species within that genus require the same or similar habitat conditions^29^. It has become a popular tool because it is a presence/pseudo-absence program and is relatively accurate with small (> 5 observations) data sets^30–33^. Despite the popularity of the tool, it is still rare to be able to subsequently validate a model with additional fieldwork, especially in an area as isolated as Vanuatu. Here, we provide results from two consecutive years of fieldwork, with the second heavily guided by the predictive model resulting from the first expedition.

Collecting efforts in both 2018 and 2019 resulted in potentially spatially biased localities because collecting areas were largely influenced by accessibility, cost, and infrastructure. When considering any sort of practical conservation, an important variable to consider is distributional data, especially with rare or endemic taxa^34^. The Wallacean shortfall makes conservation efforts difficult in general, but the problem is exacerbated with under described taxa or by accessibility to remote areas^35–38^. However, SDMs have been successful in conservation efforts within mammals^39–41^, plants^42–44^, and even insects^34,45^.

MaxEnt has been shown to be successful with species that possess both aquatic and terrestrial characteristics^25^. However, there is a large amount of variation within the coastal habitats of Vanuatu. Here we used additional field work to test the predictive power of a previously produced SDM for a coastal endemic taxon. Additionally, an updated model is presented, and results of a subsequent bias analysis are provided to add further context to the models’ interpretation.

## Methods

### Collection methods

Field expeditions were conducted in 2018 and 2019 on the islands of Ambrym, Efate, Erromango, Espiritu Santo, Gaua, Maewo, Malekula, Pentecost, and Tanna in search of *Atyphella* (Fig. 1). In 2018, specimens were found at six locations across three islands. These six presence locations were used for a preliminary MaxEnt analysis to predict areas to search for this endemic genus in the future^24^. Islands selected to be sampled in 2019 were chosen based on the Saxton et al.^24^ original model. The 2019 trip resulted in four additional localities from Efate, Espiritu Santo, Maewo, and Malekula being added to the original 2018 dataset. Only four of the islands were checked both years (Efate, Espiritu Santo, Malekula, and Tanna) which allowed for three new islands: Ambrym, Maewo, and Pentecost to be sampled in 2019. Three to five locations were checked on each of these new islands. These sites amounted to approximately five to seven kilometers of coastline on each island.

### Ecological niche model

The 30 s bioclimatic variable files accessed from WorldClim^46,47^ and the base map were trimmed and converted to ASC files using DIVA^48^. MaxEnt version 3.3.3k^49^ was run with default settings and auto features. All of the bioclimatic variables available on WorldClim were included, and a jackknife was run to help handle and visualize the correlation between the variables. The jackknife was also used to measure the variable importance. A random selection from 10,000 background grid cells was used. The default prevalence was set to 0.5, the convergence threshold was set to 0.00001, the regularization multiplier was set to one, and cross validation was used in the repeated runs. The output format was cloglog and the model was evaluated using the threshold independent area under the receiver operating characteristic curve (AUC)^26,50^. The AUC is a summary statistic to show how well a classifier, in this case a MaxEnt model, is at correctly distinguishing between a true presence and pseudo-absence location^26^. Since MaxEnt is a presence only program, it uses a variation on this technique. Instead of using known absence locations, it uses randomly selected background points—any point within the study area not marked as present—also known as pseudo-absences^26,51^. Therefore, the interpretation of the AUC is “the probability that a randomly chosen presence location is ranked higher than a randomly chosen background point”^50^. Because it uses pseudo-absences the maximum AUC is always less than one^26^. An AUC of 0.5 shows that it is no better at ranking the presence locations above the background locations^26, 51^. The higher the AUC value the better it is at minimizing either the false positives or negatives.

The WorldClim raster layers of the top contributing factors for the 2018 and 2019 models (Fig. 4) were viewed in QGIS Zanzibar 3.8^52^. The raster layers were then overlayed with occurrence points. The values associated with a known occurrence location were extracted using the identify features tool to show the variation within a factor across these locations in an attempt to explain the differences in the AUC values. All three (2018, 2019, combined) MaxEnt predictions were uploaded into QGIS to visualize how the areas predicted as suitable changed depending on different cut off values and the overlap between the predicted area and biasing factors (Fig. 7). In the cloglog output format the relative probability equates to the projected habitat suitability^53^. The probabilities were binned into ten sections with the upper probability included in that bin (e.g., 0.2–0.3). The area considered suitable in each bin was then estimated using the raster calculator. These new binary raster layers were created from just the values within each of the bins. The unique values report was then used to provide a summary of all the presence and absence pixel counts for each bin. These pixel counts were used to find the total area and a proportion within the entire archipelago for each bin (Fig. 5, Supplementary Table S2).

### Investigation of collecting bias

Shapefiles of potential biasing factors (e.g., roads and settlements) in Vanuatu were obtained from NextGIS (https://data.nextgis.com/en/region/VU/, data accessed March 2020). The bias estimation was run in RStudio 4.02^54^ through the calculate bias function from the *sampbias* package version 1.0.4^55^. The package created a grid from the border we supplied (shapefile of Vanuatu) and the distance from each cell to the nearest biasing factor was found^55^. We increased the precision of the distance estimation by setting the resolution to 0.1 (11 km^2^). The function used a Poisson sampling process to estimate the number of expected occurrences and a Bayesian approach to estimate the weight of each biasing factor^55^.

## Results

### Additional records

During 2019 the known range for *Atyphella* was expanded. One additional location was added on each of the following islands: Espiritu Santo, Malekula, and Efate (Fig. 1, Supplemental Table S1). The majority of known presence locations on Efate, Espiritu Santo, and Malekula from 2018 were checked in 2019 and all still contained *Atyphella. Atyphella* was also collected on Maewo which is the first record from the eastern islands in the Malampa and Loyalty Basin bioregion.

### Updated ecological niche model

**Figure 3:**
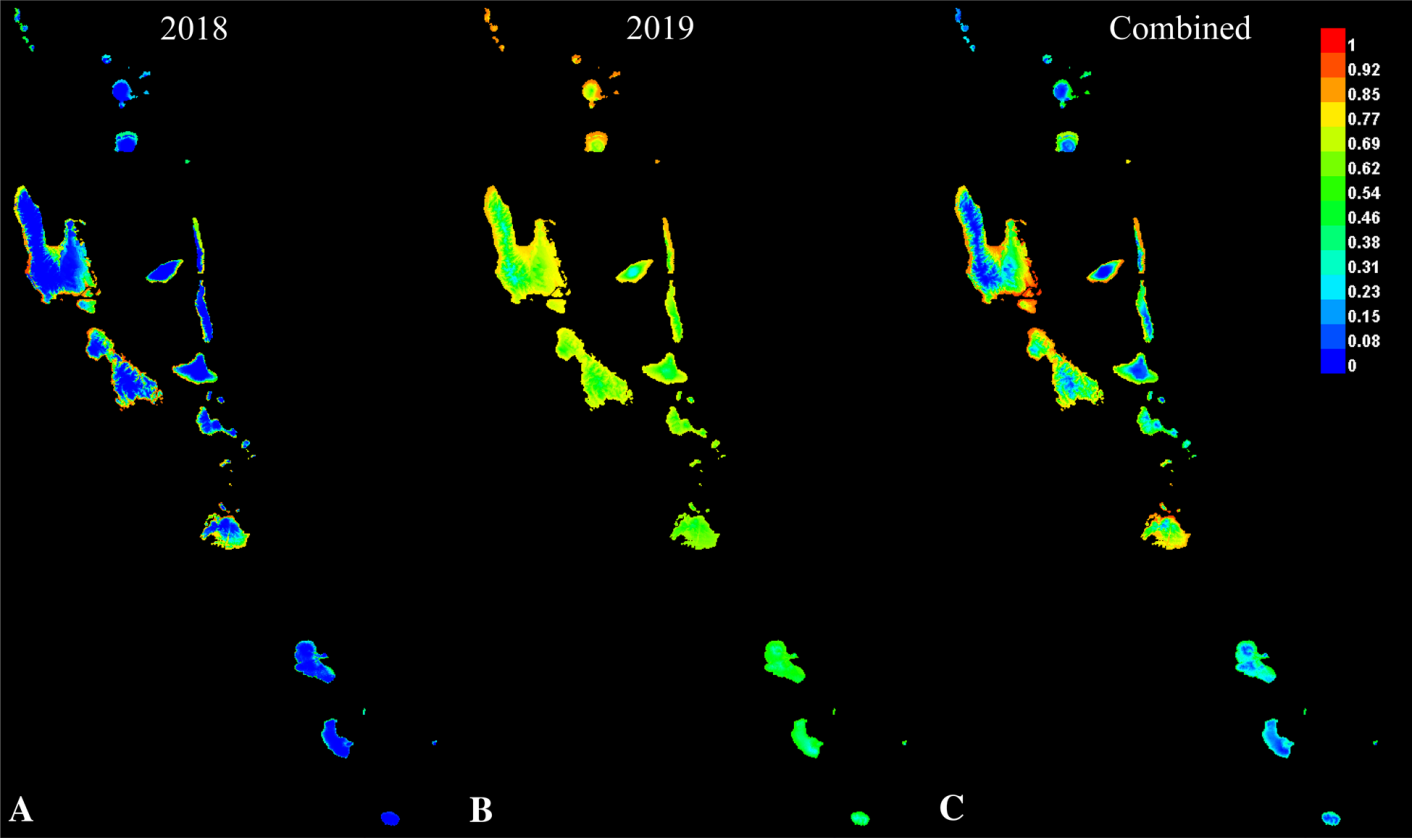
Results from MaxEnt analysis based on collection records from (**A**) only 2018 collecting, (**B**) new locations from 2019, (**C**) combined collecting events (MaxEnt version 3.3.3 k, Adobe, San Jose, CA, USA).

**Figure 4:**
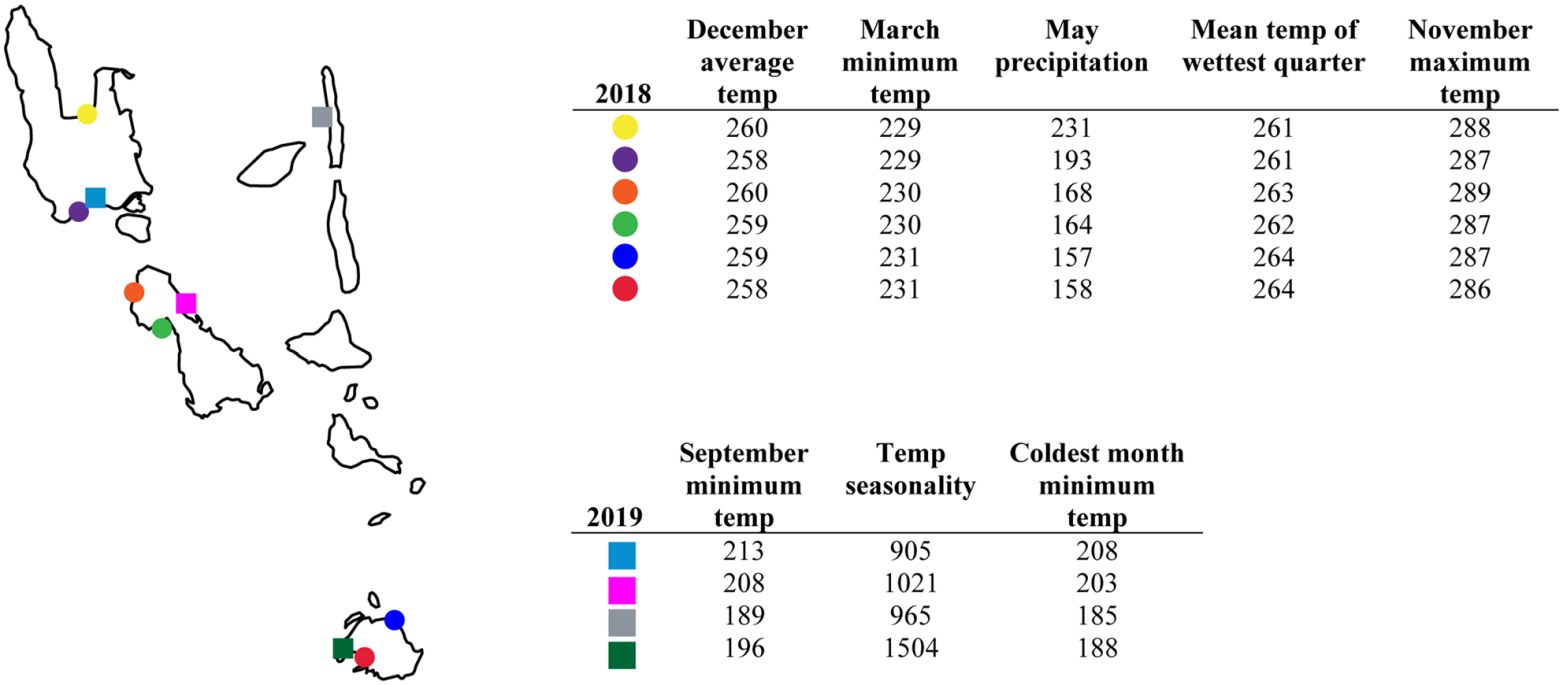
Values for the top contributing factors at each location (temperature values given in °C * 10 and precipitation values give in mm). The range of values within 2018 is minimal for each factor. There is a wider range in the values for each factor in 2019 (Vemaps.com, Adobe, San Jose, CA, USA).

Three MaxEnt outputs were generated (Fig. 3) and the highest contributing variables used in model training are reported (Table 1). These factors are shown to be much more variable in 2019 than 2018 (Fig. 4). In 2018, the range within the December average temperature and March minimum temperature values across the occurrence sites is 2 °C * 10. There is effectively no variation within these values at the occurrence sites. In 2019, there is a difference of 24 °C * 10 within the September minimum temperature values and a difference of 599 °C * 10 within the temperature seasonality values. This stark increase in variation is shown to weaken the overall model and explains the observed result which is a prediction that lacks precision or high support (Fig. 3).

**Table 1:** Comparison of major contributing factors to each MaxEnt model.

Variable	Contribution (%)
**2018**	
December average temperature	31.9
Mean temperature of wettest quarter	20.8
May precipitation	9.4
March minimum temperature	5.8
**2019**	
September minimum temperature	41.3
Temperature seasonality	32.9
Minimum temperature of coldest month	18.2
October minimum temperature	4.1
**Combined**	
August maximum temperature	21.1
Temperature seasonality	16.5
September minimum temperature	7.8
March average temperature	6.4

Temperature and precipitation are frequently used in model training in both aquatic and terrestrial systems, we found this to be the case as well. Since both coastal species of *Atyphella* from Vanuatu spend the majority of the time in the intertidal zone submerged by water, precipitation is not as influential if they were a completely terrestrial species.

**Figure 5:**
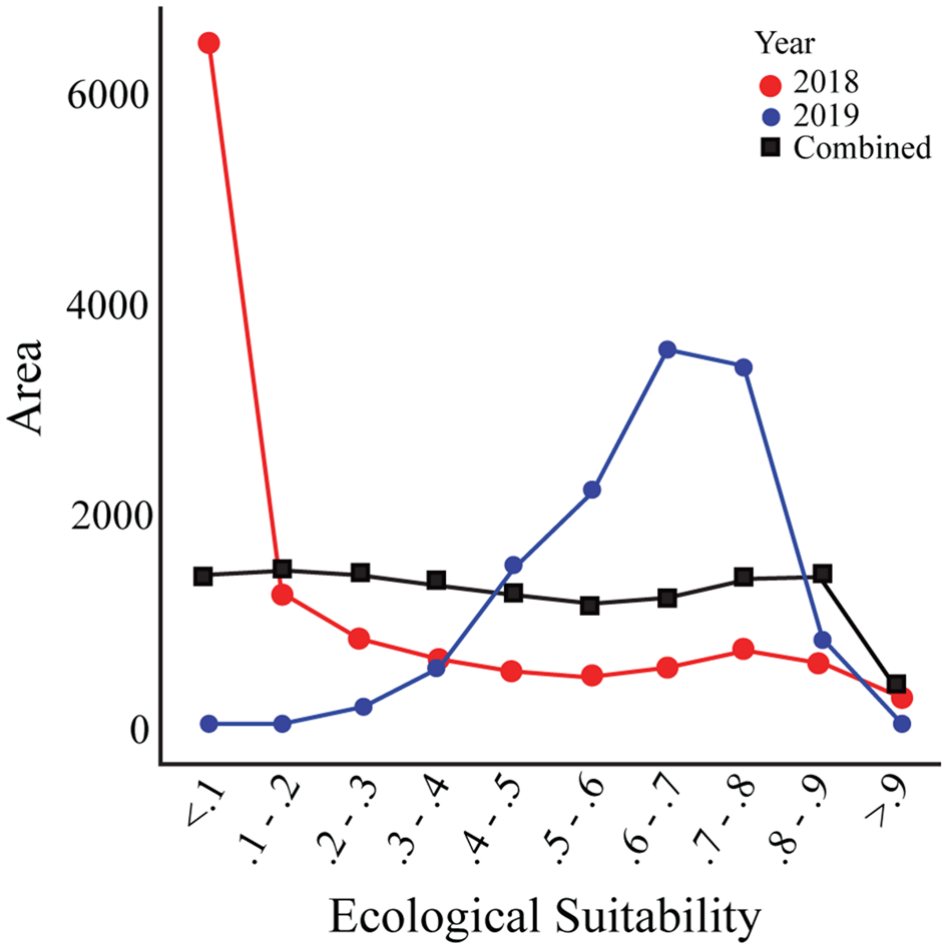
Line graph showing area of islands (in km^2^) predicted to be suitable for the presence of *Atyphella* at 10% intervals (upper probability included in interval).

The first model based on the six locations from 2018 had an AUC of 0.93 and highlighted coastal areas within the Malampa and Loyalty Basin and East Shefa bioregions^23^. The model using the four locations from 2019 had an AUC of 0.69 and the output did not show extreme probabilities (< 0.2 or > 0.8). The combined model using ten locations resulted in an AUC of 0.86 and the areas with the highest probability are along the coasts of the islands north of Chessman’s line. The amount of area within each probability is provided (Fig. 5, Supplementary Table S2). The lines for the 2018 and combined models share the same general trend which is as the probability increases the area decreases at a constant rate. For the 2019 model, as the probability increases, the area increases until 0.7, when it switches and begins to quickly decrease.

### Collecting bias

**Figure 6:**
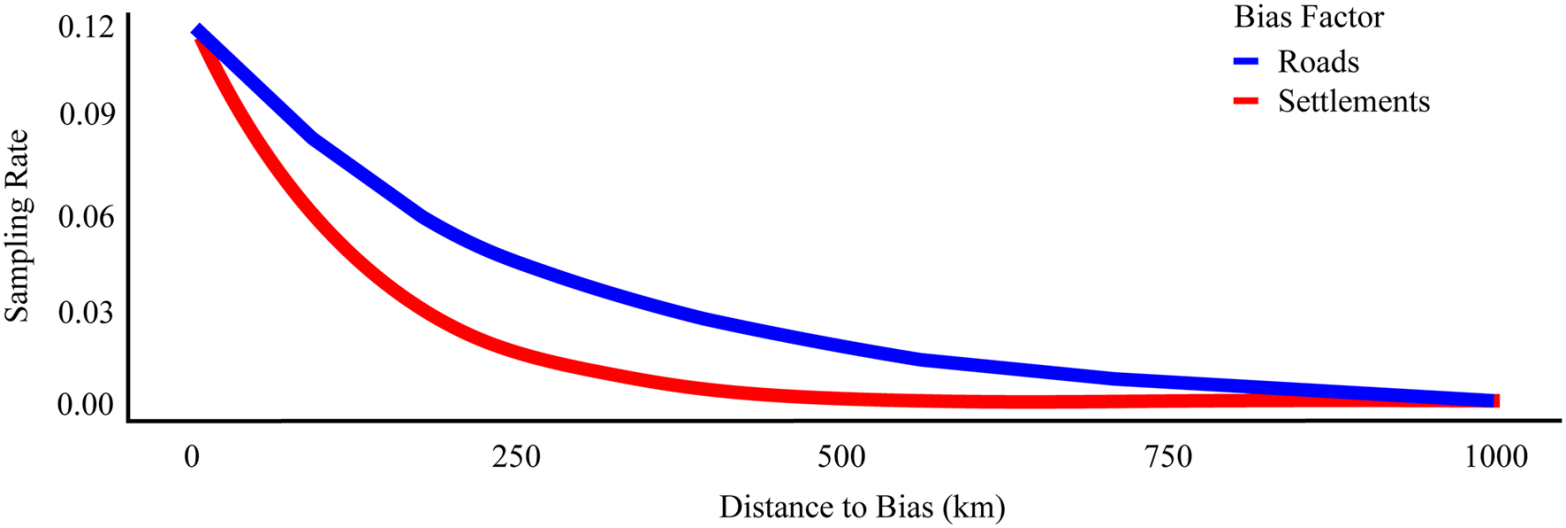
Results of *sampbias* analysis for roads and settlements. The sampling rate is the function of distance to the nearest bias factor. As the distance from a bias factor increases the less likely the location is to be sampled. Negative slopes indicate which factors are in fact biasing collecting locations with steeper slopes having more of an influence.

**Figure 7:**
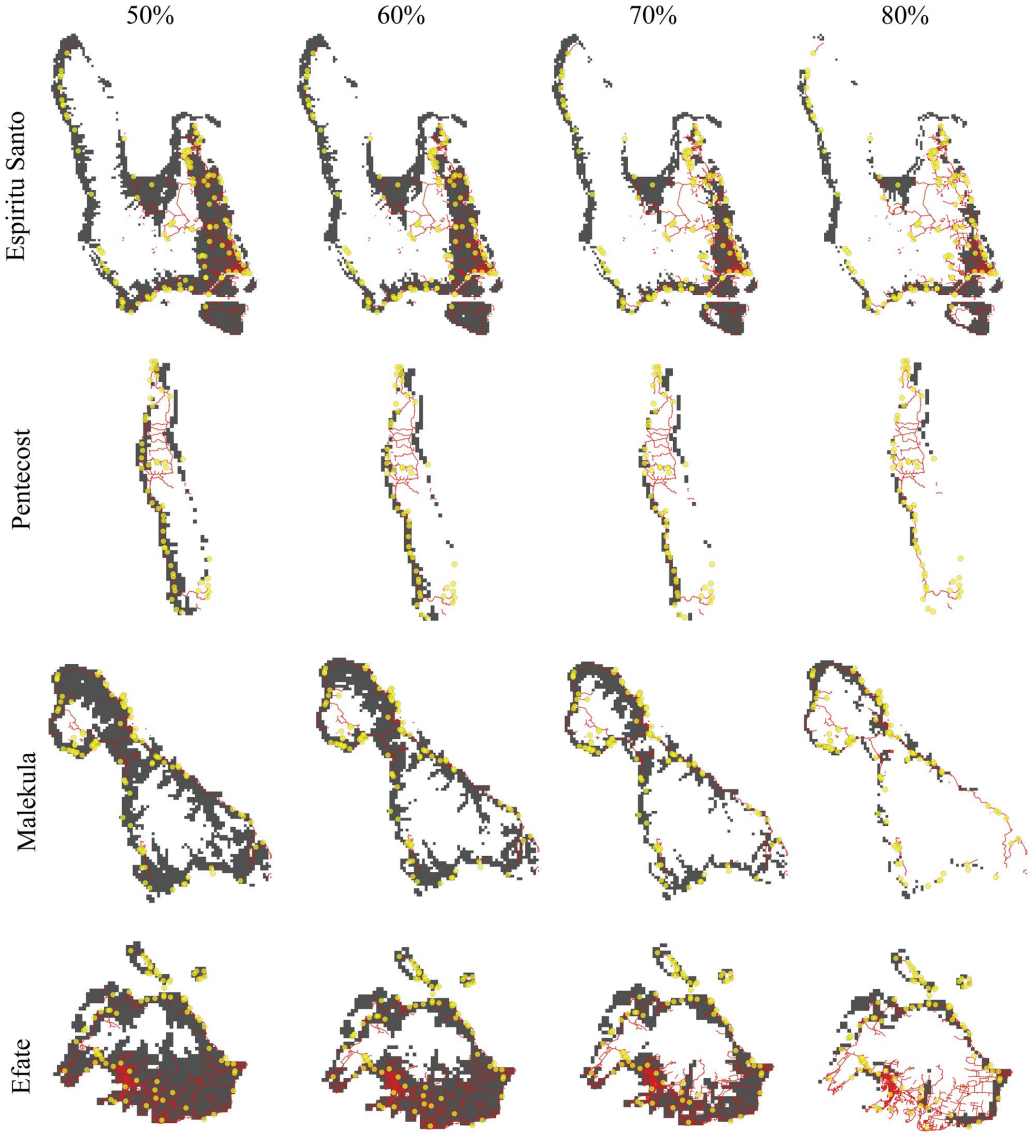
Presence of *Atyphella* predicted by MaxEnt overlaid with potential bias factors: roads (red), settlements (yellow). Rows are four heavily sampled islands, columns are thresholds of predictive results (i.e., 0.6 are those areas predicted to have *Atyphella* with > 0.6 probability in the combined model) (MaxEnt version 3.3.3 k, QGIS Zanzibar 3.8, NextGIS: https://data.nextgis.com/en/region/VU/, Adobe, San Jose, CA, USA).

The output from the *sampbias* package showed that both roads and settlements affected our sampling rate because they each have a negative slope (Fig. 6). As the distance from these factors increased the less likely that area was to be sampled. This can be seen when overlaying these biasing factors on MaxEnt outputs (Fig. 7).

## Discussion

### Ecological niche model

The 2018 MaxEnt model successfully identified new locations that were confirmed with subsequent 2019 fieldwork, thus, expanding the known distribution of *Atyphella*. This adds to the growing literature supporting the utility of MaxEnt with limited locality data (e.g.,^56–58^). The 2018 prediction correctly identified novel locations, the subsequent combined model then refined the overall range. We now have an improved understanding of their range based on the amount of time spent searching and the amount of land that we were able to cover relative to the size of the islands, approaching maximum accuracy potential as discussed by Hernandez et al.^30^. The increase in distributional data for *Atyphella* in Vanuatu has begun to minimize the negative effects associated with the Wallacean shortfall which will allow for more confidence in conservation planning. Combining data from the two years led to an increase in predictive power without a corresponding loss of precision of the model (both AUC values over 0.8) which is a result of increasing the number of sample locations and thus accuracy^31, 58–60^. Accuracy for models based on limited locations has also been shown to increase if the species requires specific habitats^30,58^. Every location that we provided was critical to the model’s training because of the variation between the most contributing factors (Fig. 4). We were able to capture the vast majority of this variation because we sampled such a wide range of coastline across the islands.

There are instances where MaxEnt predictions have been ground tested (e.g.,^61–63^), however most of these studies were conducted in more easily accessible areas. Additionally, there are studies where MaxEnt has been used to promote conservation in islands settings^43,64–66^. The models of both Kumar and Stohlgren^43^ and Thorn et al.,^66^ were limited to Borneo, Raxworthy et al.,^65^ to Madagascar, and Greaves et al.^64^ to the southern island of New Zealand, large single islands. The SDMs from these studies do not deal with challenges that come with predicting and surveying over multiple islands with varying amounts of isolation from each other. Also Kumar and Stohlgren^43^ and Thorn et al.^66^ considered a small sample to be greater than ten, we were able to successfully validate the utility of Maxent models over many islands with less data, by successive years of field research. This study focused on remote locations with extremely rare endemic species with very specific habitats and was shown to have tremendous value and accuracy.

The 2019 model generated from only four locations is less predictive because the number of locations for consistent “significant predictive ability” is five or above^32^. Combining data from these models led to a much more robust result, as expected. It is also accepted that outliers and additional records carry significant weight and can influence the prediction when working with small sample sizes if these new locations provide different environmental variables or values^30,32^. Our 2019 data when analyzed alone illustrated this issue. However, when combining all available data this problem was mitigated by including the full breadth of observed variation.

### Collecting bias

There are numerous issues that arise when working with distributional data (i.e., the Wallacean shortfall), but efforts and recommendations are being made to mitigate these effects^35,67^. Distribution data is essential for studying global patterns of diversity and range shifts for invasive or threatened species, but the extent to which it is collected is inconsistent^36,68^. Museum collections are often a combination of preferential collecting and intense surveying of certain areas which can bias research results^68^. Steps are being taken to digitize and combine museum records to allow for broader access and likely leading to more accurate analyses^67–69^. While access to more data is beneficial, it is the quality of the records that need to be assessed^67^. Most studies are very scale dependent. For instance, worldwide patterns can be seen with very course data (e.g., country records) while studies focused on smaller geographic areas need finer scale data (e.g., precise coordinates)^36,67^. It is difficult to get these data in remote locations where access is limited, as is the case with Vanuatu, therefore post hoc analysis can assist identifying bias when interpreting results^36^.

It is important to identify if there is spatial bias in SDMs because if unknown, the model results can be interpreted incorrectly. If the collection locations are biased and the background locations which are randomly chosen over the entire area are not biased then the results are skewed^33^. While there are a few methods to account for spatial bias (e.g., spatial filtering and background manipulation) there is no agreed upon approach^33,70^. Fourcade et al.^71^ found that the most consistent spatial filtering approach was systematic sampling. However, this simply reduces the number of locations and does not account for a lack of data, which is an issue with the dataset for *Atyphella*. As for the background manipulation approach, a buffer is created around the locations from which background locations can be chosen^71^. This does not work in Vanuatu because the size of each island is too small to create an appropriate buffer. With the extremely limited locations for *Atyphella*, we cannot use the traditional approaches to correct for spatial bias. These approaches only attempt to correct the prediction and do not identify the original causes of bias. Instead, we conducted a post hoc analysis to identify bias which is shown to work well with sparse data and small areas^55^. Identifying the biasing factors allows us to consider potentially affected areas, and make more informed conclusions, better guiding conservation efforts.

**Figure 8:**
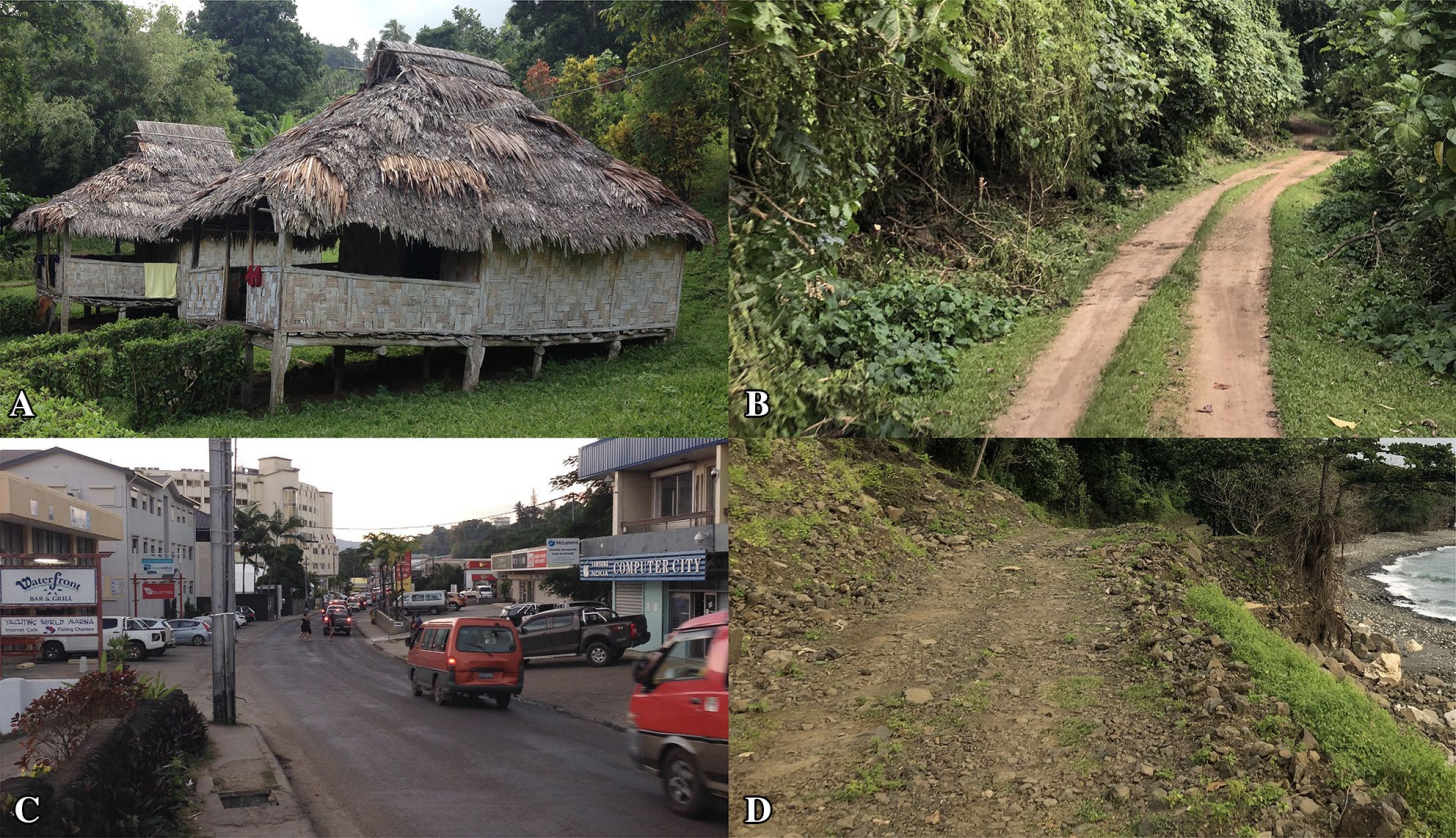
(**A**) Example of settlements on more remote islands. (**C**) Example of settlements and roads on more developed islands. (**B**–**D**) Variation within roads across the islands (Photo credit: Colin Jensen and Natalie Saxton).

The bias in the model stems from the fact that Vanuatu has relatively little infrastructure and it is quickly and constantly changing. The biasing layers available for Vanuatu are roads and settlements, but what is included as a road or settlement varies considerably between the islands. For example, on the more developed islands of Efate, Espiritu Santo, and Tanna the roads were often asphalt or concrete and settlements were often larger. On the other islands, the ‘roads’ closely resemble dirt trails and structures within the settlements are less common and built from surrounding natural materials (Fig. 8). Even though there are issues with consistency across the layers, this analysis would not have been possible a few years ago because the layers had not yet been developed. Information that will improve these data layers and expand the possibilities of research similar to ours is currently being compiled for islands across the South Pacific in response to rapid commercialization, globalization, and environmental threats^23^.

In addition to infrastructure, cost and time were the other limiting factors. During fieldwork, our research team often reached the end of a road and could continue on foot for short distances but were often limited by social, environmental, or academic factors. The majority of the roads followed the coast because the economy is centered around marine activities (e.g., fisheries) and tourism. The other reason is because the islands are volcanic and road development further inland is minimal and travel is difficult due to the topography. While analyses showed that the collecting was biased, the data that we were able to gather is still extremely valuable and we can more accurately interpret the results in order to correct for this bias in the future. There are large areas of coastline on both Espiritu Santo and Pentecost that do not have roads or settlements and these areas are not predicted to have suitable habitat. The areas on Espiritu Santo and Pentecost that are predicted to have suitable habitat also have roads and settlements nearby. Another example of this bias is seen on Efate. There were three locations on the northern half of the island where *Atyphella* was collected, so it is expected that this half would have high probabilities of suitable habitat associated with it. However, within Efate as we increased the probability of presence, the areas that remained suitable were farther south where the biasing factors were more prominent. Across the entire archipelago, as we increased the minimum threshold for probability of occurrence, the areas considered suitable were largely areas with biasing factors.

The combined model can aid Vanuatu's marine spatial planning committee, by identifying coastal locations that are important to fireflies and firefly protection. The coastlines are under serious threat from rising sea levels, tropical storms, human development, and commerce^23,72^. Gassner et al.^23^ showed that 40.9% of all reef areas are at “high risk” and 13.8% are at an “extremely high risk” due to coastal development (ports, roads, and housing), fishing activities, and human activities. The majority of these high risk areas are the same coastal areas that contain firefly populations. There are currently 14 ports across the archipelago used mainly for commercial fishing, cargo, and tourism. The largest ports are located on Efate and Espiritu Santo which are both home to coastal *Atyphella* species and have additional areas predicted to be appropriate habitat for *Atyphella* to survive. Altering the coastlines to support development lessens the natural protections they provide from tropical storms and destroys habitats for many species^23^. Other threats the coastlines are facing come from the ocean itself. Sea levels are rising which is extremely problematic for the low-lying islands of Vanuatu^72^. Gassner et al.^23^ also showed how sea levels are predicted to rise a minimum of 0.15 m by 2030. In addition, shallow water along the coastline is quickly warming and becoming more acidic (pH 8.26–8.3) which is a major concern for fireflies as both females and larvae spend a significant amount of their life-cycle in or near the water. If coastal development, ocean levels, water temperatures, and acidity continue to increase there will be significant issues for endemic species dependent on coastal habitats, such as fireflies.

Vanuatu’s government is aware of the threats facing their marine and coastal habitats. They were the first Pacific Island nation to have a National Ocean Policy which uses marine spatial planning and climate resistant networks to make policies to conserve the natural diversity within the nation^23,73^. They are also aware that the natural beauty and biodiversity of Vanuatu is a major draw for tourists. There is already a coastal resort adjacent to a known *Atyphella* habitat which has become a draw for the resort. One of the main tourism slogans is “Discover what matters” with the main attractions being diving and snorkeling and if these areas are not preserved Vanuatu’s economy will be negatively impacted^23^ (p. 49). Additionally, local leaders have been regulating the fishing industry for years to make it as sustainable as possible, and the national government is following suit in the commercial fishing industry. We can see that the government is committed to protecting what makes Vanuatu unique. One more conservation policy to protect the habitats these extremely unique fireflies need would complement the plans that the country already has in place very well.

## Supplementary Information

Supplementary Tables.

## Data Availability

Presence localities in supplementary material Table S1.
