# Supplementary material for: Validating species distribution models to illuminate coastal fireflies in the South Pacific (Coleoptera: Lampyridae)
Source: Sci Rep. 2021 Aug 30;11:17397. doi: 10.1038/s41598-021-96534-x (PMC8405826; doi:10.1038/s41598-021-96534-x)
Supplement: Supplementary file 1 — Supplementary Tables. [file 41598_2021_96534_MOESM1_ESM.pdf]

Validating species distribution models to illuminate coastal fireflies in the South Pacific (Coleoptera: Lampyridae)

Laura N. Sutherland, Gareth S. Powell, Seth M. Bybee

Year	Latitude	Longitude
2018	-15.62	166.854
	-15.1598	166.952
	-16.111	167.332
	-16.029	167.173
	-17.719	168.176
	-17.581	168.47
2019	-15.116	168.09
	-15.57	166.951
	-16.058	167.389
	-17.691	168.24

Table S1. Coordinates for all successful collection locations of *Atyphella* in 2018. Coordinates for new successful collecting locations in 2019.

**Ecological Suitability**	**Pixels**			**Area**			**Percent**		
	2018	2019	Combined	2018	2019	Combined	2018	2019	Combined
<.1	8434	0	1804	6474.49	0	1384.87	53.12	0	11.36
.1-.2	1619	1	1883	1242.85	0.77	1445.51	10.2	0.01	11.86
.2-.3	1047	185	1843	803.75	142.02	1414.81	6.59	1.17	11.61
.3-.4	805	687	1749	617.97	527.39	1342.65	5.07	4.33	11.02
.4-.5	668	1964	1617	512.8	1507.7	1241.32	4.21	12.37	10.18
.5-.6	571	2892	1444	438.34	2220.09	1108.51	3.6	18.21	9.09
.6-.7	697	4664	1533	535.06	3580.39	1176.83	4.39	29.37	9.65
.7-.8	927	4459	1786	711.63	3423.02	1371.05	5.84	28.08	11.25
.8-.9	763	1026	1816	585.73	787.63	1394.08	4.81	6.46	11.44
>.9	347	0	403	266.38	0	309.37	2.19	0	2.54

Table S2. Summary of predictive output given in pixels, area (km^2), and percentage of total landmass of Vanuatu.
